# Natural elimination of a video capsule after retention for 1 year in a patient with small bowel Crohn disease: A case report

**DOI:** 10.1097/MD.0000000000017580

**Published:** 2019-10-25

**Authors:** So Yeon Lee, Ju Yup Lee, Yoo Jin Lee, Kyung Sik Park

**Affiliations:** Department of Internal Medicine, Keimyung University School of Medicine, Dalseo-gu, Daegu, Republic of Korea.

**Keywords:** capsule endoscopy, Crohn disease, obstruction, retention, small bowel

## Abstract

**Introduction:**

Video capsule endoscopy (VCE) is a useful tool to differentiate small intestinal bleeding, inflammatory bowel disease, and other small bowel disease. The most common adverse effect of VCE is capsule retention; the incidence varies greatly depending on the underlying disease, which is known to increase from 1.5% in healthy individuals to 21% in patients with small bowel Crohn disease. We report this case on a patient who had asymptomatic capsule retention for 12 months and experienced natural elimination with medication.

**Patient concerns:**

A 21-year-old woman presented to the hospital with chronic abdominal pain and persistent diarrhea for 2 years.

**Diagnoses:**

The patient was diagnosed with small bowel Crohn disease using VCE, and radiography revealed capsule retention.

**Intervention:**

Symptoms of obstruction were not distinctive, it was decided to increase the dosages of azathioprine and infliximab to 50 and 500 mg (10 mg/kg), at 5 months after VCE. And at month 11 of capsule retention, she was admitted and started on a regimen of hydrocortisol 300 mg for 4 days and hydrocortisol injection 200 mg for 10 days.

**Outcomes:**

At month 12, abdominal radiography in the clinic confirmed that the capsule had been naturally retrieved.

**Lessons:**

Capsule retention could be initially treated conservatively with medication and if the treatment fails, it is recommended to remove the capsule surgically. But in the case of the clinical condition of the patient is favorable without symptoms of bowel obstruction, the medication should be continued and the patient followed up.

## 1 Introduction

Video capsule endoscopy (VCE) is a useful tool to differentiate small intestinal bleeding, inflammatory bowel disease, and other small bowel disease. Capsules are typically retained for 24 to 48 hours and then naturally eliminated; capsule retention is reported to have a low incidence of adverse effects. Moreover, VCE is reported to be more effective than computed tomography (CT) enterography and magnetic resonance enterography for confirming lesions in the proximal small bowel.^[1]^ However, biopsy cannot be performed with VCE and the test procedure is limited in 15% to 30% of patients. The overall incidence of capsule retention is reported to be 2.6%, although the rate increases to 21% in patients with small bowel Crohn disease.^[2]^ There have been cases in which capsule retention caused a complication, such as small bowel perforation, within several days,^[3]^ but cases without symptoms or complications have also been reported. The longest time of capsule retention without a symptom or complication reported in the literature is 7 years.^[4]^ The European Society of Gastrointestinal Endoscopy (ESGE) recommends that capsule retention can be initially treated conservatively with medication and then additional treatment, such as enteroscopy, be performed if natural elimination fails. If enteroscopy fails, it is recommended to remove the capsule surgically, but if the clinical condition of the patient is favorable without symptoms of bowel obstruction, the medication should be continued and the patient followed up.^[5]^ Here, we report a patient who presented to the hospital with chronic abdominal pain and was diagnosed with small bowel Crohn disease using VCE. The capsule was retained for 12 months and naturally retrieved with medication. The patient has provided informed consent for publication of the case.

## 2 Case

A 21-year-old woman presented to the hospital with chronic abdominal pain and persistent diarrhea for 2 years. Small bowel Crohn disease was suspected based on abdominal CT findings and she was transferred to a tertiary hospital. She had no notable underlying disease or family disease history. She scored her abdominal pain as 4 to 5 on a visual analog scale. The pain occurred mainly at night and was accompanied by watery diarrhea 2 to 4 times per week. She experienced weight loss of approximately 30 kg over the past 2 years.

On admission, her blood pressure was 130/80 mm Hg, respiratory rate was 18 breaths/min, pulse rate was 75 beats/min, and temperature was 36.7°C. She exhibited chronic systemic weakness but was fully conscious. On physical examination, no abnormal findings in the conjunctiva or sclera were observed. Bowel sounds were normal but pressure pain was confirmed in the lower abdominal area bilaterally. No rebound tenderness or abdominal cysts were found. No lesions on the skin were observed and no swollen lymph nodes in the neck or axillary region were found.

Laboratory finding revealed normal leukocyte count, liver and renal function test. Only C-reactive protein increased to 2.12 mg/dL (normal, 0–0.5 mg/dL) and erythrocyte sedimentation rate increased to 43 mm/h (normal, men: <15 mm/h; women: <25 mm/h).

Colonoscopy revealed multiple superficial ulcers and nodular mucosa in the terminal ileum and an aphthous ulcer in the sigmoid colon. Biopsy of tissue samples from the aphthous ulcer site in the sigmoid colon confirmed mucosal ulcer and granuloma. Abdominal CT performed at the first hospital showed edema and mucosal thickening in the small bowel but no finding of distinctive stenosis. Additionally, a comb sign was present, suggesting the development of bifurcated vessels in the small bowel, confirming an active phase of Crohn disease. VCE was performed to determine the extent and severity of the lesion in the small bowel, and a diagnosis of small bowel Crohn disease was made based on findings of multiple circular ulcers in the proximal jejunum and localized luminal stenosis. Her Crohn disease activity index (CDAI) was 300, indicating moderate activity. She was prescribed prednisolone 40 mg, azathioprine 25 mg, and 5-aminosalicylic acid 3 g.

**Figure 1:**
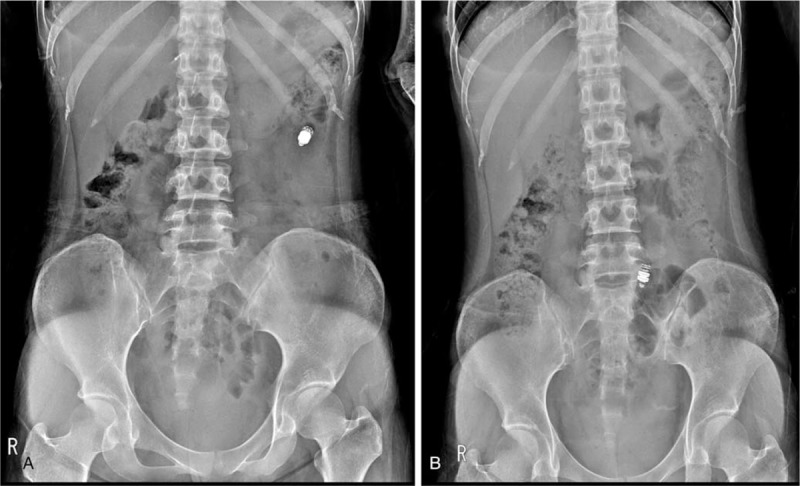
Serial abdominal radiographs after capsule endoscopy. (A) Radiograph 2 weeks after capsule endoscopy shows capsule retention in the left upper quadrant. (B) Radiograph 3 months after capsule endoscopy shows capsule retention in the lower abdomen.

Radiography 2 weeks later revealed capsule retention near the proximal jejunum (Fig. 1A). Based on the absence of distinctive stenosis on preprocedure CT, it was expected that the capsule would be naturally retrieved if her Crohn disease activity was under control. Thus, the medication was continued and the patient followed up.

One month later, her symptoms had not improved and infliximab 250 mg (5 mg/kg) was added to the regimen. At month 3, her symptoms had improved and her CDAI decreased to 180, suggesting improvement in disease activity. However, radiography still showed capsule retention in an area estimated to be near the small bowel and ileum toward the pelvis (Fig. 1B). She continued the medication regimen. Infliximab infusion was administered once every 8 weeks, but she had very low adherence to the oral drugs.

**Figure 2:**
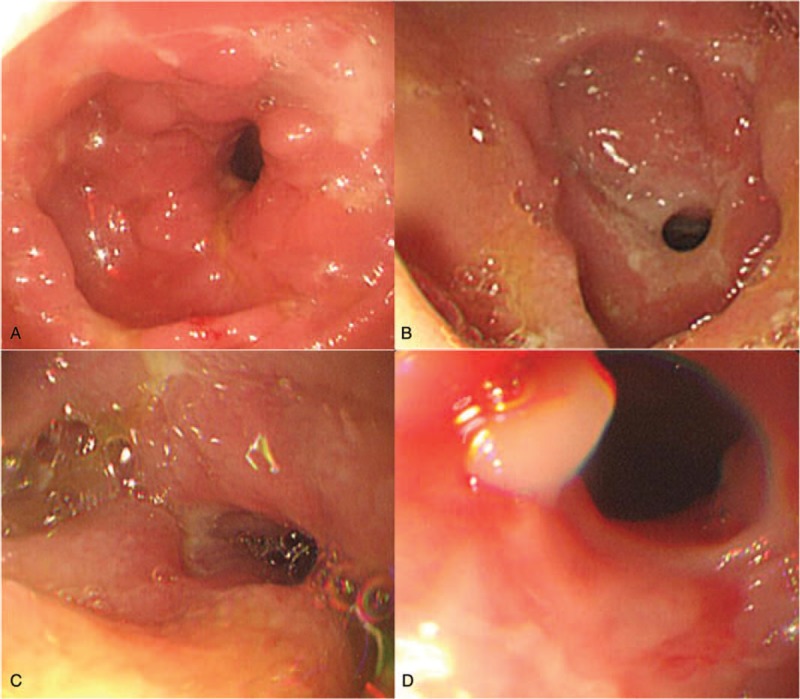
Single-balloon enteroscopy images show multiple areas of luminal narrowing and mucosal deformity with a cobblestone appearance.

Five months after VCE, her abdominal pain worsened. CT enterography showed capsule retention near the stenosis in the ileum and enlargement of the small bowel proximal to the stenosis. It was determined that the stenosis was too severe for the capsule to be naturally retrieved and single-balloon enteroscopy was performed. During the procedure, multiple areas of stenosis were found in the jejunum and it was impossible to pass the enteroscope through the proximal ileum (Fig. 2). Thus, enteroscopic capsule removal was failed because the enteroscope could not reach the location of capsule retention. Although the operation was considered because enteroscopy was failed, her obstructive symptoms were not distinctive relative to her pain, thus it was decided to increase the dosages of azathioprine and infliximab to 50 and 500 mg (10 mg/kg), respectively. Subsequently, her abdominal pain was alleviated.

**Figure 3:**
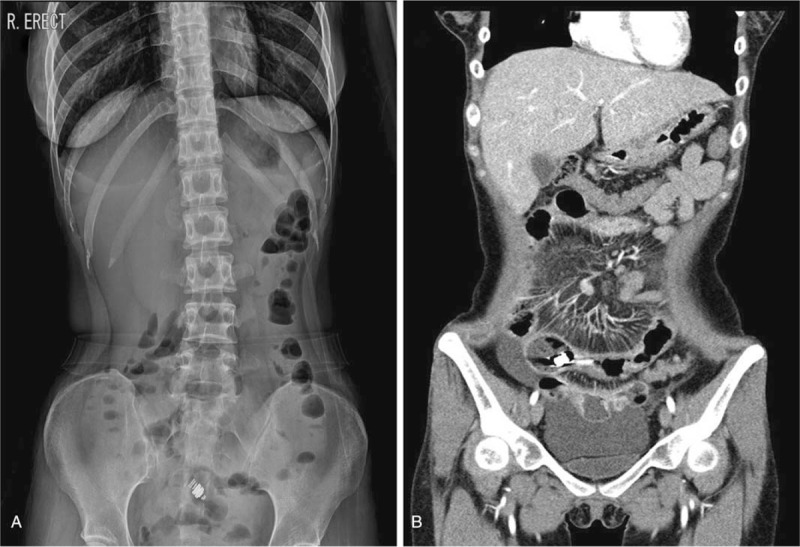
Abdominal radiograph and computed tomography scan 11 months after capsule endoscopy. (A) Stepladder sign and capsule retention are identified in the lower abdomen. (B) Multifocal wall thickenings and luminal narrowing are observed in the ileum, and capsule retention is identified near the luminal narrowing.

At month 11 of capsule retention, she returned to the hospital due to worsening of abdominal pain. Abdominal radiography showed a stepladder sign, suggesting bowel obstruction, and CT revealed multiple areas of stenosis and capsule retention in the lumen of the distal ileum (Fig. 3). She was admitted and started on a regimen of hydrocortisol 300 mg for 4 days and hydrocortisol injection 200 mg for 10 days after being informed that resection would be necessary if fasting and steroid injection were ineffective. Subsequently, her symptoms of bowel obstruction and imaging findings improved and she was discharged.

**Figure 4:**
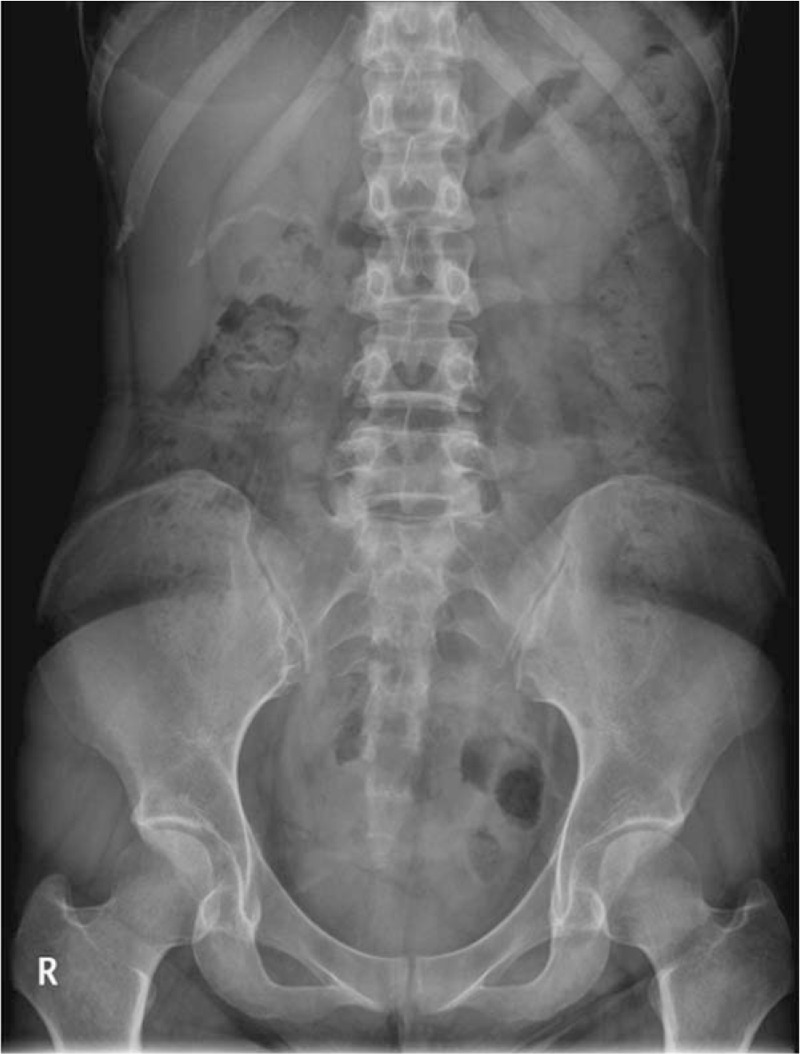
Radiograph 12 months after capsule endoscopy shows disappearance of the capsule.

At month 12, abdominal radiography in the clinic confirmed that the capsule had been naturally retrieved (Fig. 4). At present, her bowel symptoms are improved and she is being followed up as an outpatient.

## 3 Discussion

VCE is a noninvasive and simple procedure that currently plays an important role in examining and diagnosing lesions in the small bowel. A capsule is typically sized approximately 1 × 2.5 cm and contains a camera, battery, light, and component for image transmission. Images are transmitted to a sensor attached to the patient's body for 8 hours after capsule ingestion. In most cases, the capsule is eliminated with feces within 10 to 48 hours.^[6]^

Indications for VCE include suspected small intestinal bleeding, small bowel Crohn disease, small bowel tumor, and malabsorption syndrome. However, VCE use is prohibited in patients with suspected gastrointestinal stenosis, fistula, or obstruction; patients using a pacemaker; and patients with a swallowing disorder.^[7]^ The most common complication of VCE is capsule retention. In 2005, the International Conference on Capsule Endoscopy defined capsule retention as the retention of a capsule in the gastrointestinal tract for at least 2 weeks or requiring enteroscopic or surgical intervention aside from medication for removal.^[6]^ The incidence of capsule retention is reported to vary greatly depending on the underlying disease, from 1.5% in healthy individuals to 21% in patients with small bowel Crohn disease.^[8]^ According to a study by Cheon et al, the incidence of capsule retention is higher in small bowel Crohn disease compared with other types of small bowel disease.^[9]^ Also, the likelihood of capsule retention increases in patients with nonsteroidal anti-inflammatory-induced enteritis, tuberculous enteritis, ischemic enteritis, radiation enteritis, and stenosis following surgery.^[2]^ Hence, to prevent capsule retention, it is important to carefully take patients’ disease and medication histories before performing VCE.^[6]^ The ESGE also recommends imaging studies using a contrast agent before performing VCE. The guideline states that it is important to confirm the presence or absence of luminal stenosis, particularly if the patient suffers from abdominal pain or has symptoms of bowel obstruction.^[10]^

Capsule retention is asymptomatic in most cases, but partial bowel obstruction can occur in some patients. According to a study by Tang et al, 85% of patients with capsule retention were asymptomatic and the remaining 15% presented with symptoms of bowel obstruction, such as abdominal pain and vomiting.^[11]^ ESGE guidelines state that patients with capsule retention who are in favorable condition without symptoms of bowel obstruction may naturally retrieve the capsule if initially treated with medication, such as anti-inflammatory agents and immunomodulators.^[5]^ According to Fernández-Urién et al 64.4% of 104 patients with capsule retention were treated by nonsurgical methods. Of these, 19.2% of patients were able to remove the capsules using steroids or laxatives.^[12]^ If the capsule is not removed with medication, endoscopy may be attempted. Bhattarai et al reported a case of 4 years and 5 months asymptomatic capsule retention in a 49-year-old man who had subtotal colectomy with ulcerative colitis, which was resolved by colonoscopy procedure.^[13]^ If endoscopy is not successful, a small number of patients may require surgical intervention, whereas asymptomatic patients may initially be put under observation.^[14]^ However, simple follow-up in asymptomatic patients does not always produce good results. Palmer et al reported a case involving a 62-year-old woman with small bowel Crohn disease who underwent resection because 48 hours after VCE, small bowel perforation occurred in the area proximal to capsule retention.^[3]^ Cátia et al reported a case in which surgical intervention was performed to remove a capsule in a patient with small bowel diverticulitis who presented with symptoms of bowel obstruction after being followed up for 7 years for asymptomatic capsule retention.^[15]^ In such cases, perforation may occur within a week or a few years, so careful attention and periodic follow-up are necessary even in the absence of symptoms.

We report this case with a review of literature on a patient with small bowel Crohn disease who had asymptomatic capsule retention for 12 months and experienced natural elimination with medication.

## Author contributions

**Conceptualization:** Ju Yup Lee.

**Funding acquisition:** Ju Yup Lee.

**Supervision:** Yoo Jin Lee, Kyung Sik Park.

**Writing – original draft:** So Yeon Lee.

**Writing – review and editing:** Ju Yup Lee.

Ju Yup Lee orcid: 0000-0003-0021-5354.
